# Association between precarious employment and the onset of depressive symptoms in men and women: a 13-year longitudinal analysis in Korea (2009–2022)

**DOI:** 10.1017/S2045796024000258

**Published:** 2024-04-16

**Authors:** Seong-Uk Baek, Jong-Uk Won, Yu-Min Lee, Jin-Ha Yoon

**Affiliations:** 1 Department of Occupational and Environmental Medicine, Severance Hospital, Yonsei University College of Medicine, Seoul, Korea; 2 The Institute for Occupational Health, Yonsei University College of Medicine, Seoul, Korea; 3 Graduate School, Yonsei University College of Medicine, Seoul, Korea; 4 Department of Preventive Medicine, Yonsei University College of Medicine, Seoul, Korea

**Keywords:** depression, employment condition, employment precariousness, employment quality, mental health, social determinant of health

## Abstract

**Aims:**

Increasing social concern surrounds the potential adverse health effects of precarious employment (PE). In this study, we explored the association between PE and the onset of depressive symptoms.

**Methods:**

A total of 11,555 Korean waged workers (5700 females) contributed 62,217 observations from 2009 to 2022. PE was operationalized as a multidimensional construct, including employment insecurity, income inadequacy and lack of rights and protection. Depressive symptoms were evaluated using the Center for Epidemiological Studies-Depression Scale (11-item version). The association between PE and the onset of depressive symptoms in the subsequent year was estimated using generalized estimating equations. Effect sizes were reported as odds ratio (OR) and 95% confidence interval (CI).

**Results:**

The overall incidence of depressive symptoms was 8.3% during the study period. In cross-sectional analysis, daily employment, disguised employment, lower monthly wages and lack of social insurance coverage were associated with concurrent depressive symptoms in both men and women. Longitudinally, fixed-term employment (OR: 1.17, 95% CI: 1.07–1.29), daily employment (OR: 1.64, 95% CI: 1.45–1.85) and disguised employment (OR: 1.36, 95% CI: 1.17–1.57) were associated with the onset of depressive symptoms among the overall sample. Among men, the lowest quartiles of wage were associated with the onset of depressive symptoms (OR: 1.34, 95% CI: 1.13–1.60), while the absence of a trade union was associated among women (OR: 1.18, 95% CI: 1.01–1.39).

**Conclusions:**

Employment insecurity, inadequate income and lack of rights and protection may contribute to depressive symptoms. Therefore, PE serves as a significant social determinant of mental health among workers in Korea. Active policy efforts are warranted to improve the overall quality of employment in the workforce.

## Introduction

Precarious employment (PE) is acknowledged as a significant social determinant of health. Although lacking a universal definition, the existing literature recognizes PE as encompassing various employment conditions, such as employment insecurity, inadequate income and the absence of worker rights and protection (Bodin *et al.*, 2020; Kreshpaj *et al.*, 2020). PE is disproportionately distributed across socio-demographic backgrounds, including gender, age, educational level and immigrant status, thereby contributing to the exacerbation of health inequalities (Murillo-Huertas *et al.*, 2023). Recent transformations in the global labour market, such as the Fourth Industrial Revolution or the ‘gig’ economy, have the potential to increase the prevalence of PE among workforces.

Previous studies have associated PE with various adverse health outcomes, including poor self-rated health (Pulford *et al.*, 2022), cardiovascular diseases (Matilla-Santander *et al.*, 2022) and all-cause mortality (Matilla-Santander *et al.*, 2023). Alongside physical conditions, PE is closely associated with mental health. Systematic reviews have demonstrated that specific elements, such as temporary employment or perceived job insecurity, exhibit positive associations with depressive symptoms or psychological distress (Jaramillo *et al.*, 2022; Ronnblad *et al.*, 2019). In recent years, studies have increasingly focused on the multi-dimensionality of PE. For instance, Swedish studies have shown that those experiencing low-quality employment, characterized by insecure employment, low income and lack of rights and protection, have an increased risk of developing common mental disorders, suicidal behaviours and substance dependence (Jonsson *et al.*, 2021b; Pollack *et al.*, 2022). Furthermore, a cross-sectional study in Korea revealed that multifaceted aspects of PE, including temporary employment, low wage and lack of trade union representation, are closely related to depressive symptoms and sleep disturbance (Baek *et al.*, 2023; Gong and Park, 2023). Regarding the mechanism linking the relationship between PE and mental health, chronic stress plays a significant role. For instance, recent studies have shown that individuals with high levels of employment precariousness experience elevated levels of stress (Julia *et al.*, 2022; Mendez-Rivero *et al.*, 2022), which may act as a trigger for the development of depression and anxiety. The heightened stress experienced by workers in PE may stem from the combination of demanding job roles and insufficient resources or control to manage them effectively (Bakker and Demerouti, 2007; Rivero *et al.*, 2021).

Despite the increasing scholarly interest in the relationship between PE and mental health, the current body of literature has limitations. First, previous studies on the impact of PE on mental health were predominantly based on European contexts, with limited representation from East Asian countries such as Korea. Considering that the manifestation of PE and its influence on mental health can be greatly influenced by regional policies (Padrosa *et al.*, 2022), its association with the onset of depressive symptoms among Korean workers can provide a meaningful contribution to the literature. Second, as highlighted in previous systematic reviews (Jaramillo *et al.*, 2022; Ronnblad *et al.*, 2019), studies examining the relationship between PE and depressive symptoms often relied on a unidimensional definition based on the contract type; the association between depressive symptoms and PE elements beyond temporary employment remains an understudied area (Jaramillo *et al.*, 2022; Ronnblad *et al.*, 2019). Third, previous Korean studies on the effects of PE on mental health have predominantly utilized a cross-sectional design (Baek *et al.*, 2023; Gong and Park, 2023), thus failing to establish a temporal association between PE and depressive symptoms. Therefore, this study aims to explore the association of PE and the onset of depressive symptoms among Korean workers by encompassing its multidimensional aspects, including employment insecurity, income adequacy and lack of rights and protection.

## Methods

### Study sample

The study sample was derived from the Korean Welfare Panel Study (KWPS), a longitudinal survey conducted annually since 2006 by the Seoul National University Institute of Social Welfare and Korea Institute for Health and Social Affair. In the initial wave of the KWPS, a total of 7072 households and their members, representative of the Korean population, were included through systemic sampling. Trained interviewers conducted surveys through household visits for each selected household. The sample participation rate experienced an annual decline of about 3–5%, with 5853 households surveyed in the final wave (Wave 17, 2022). An additional 1800 and 2012 households were incorporated into the study sample in 2012 and 2022, respectively to address sample attrition. After including households formed through the separation of existing households, for example, when adult offspring create a new household, a total of 11,204 households and 24,077 individuals were surveyed during the study period.

**Figure 1.**
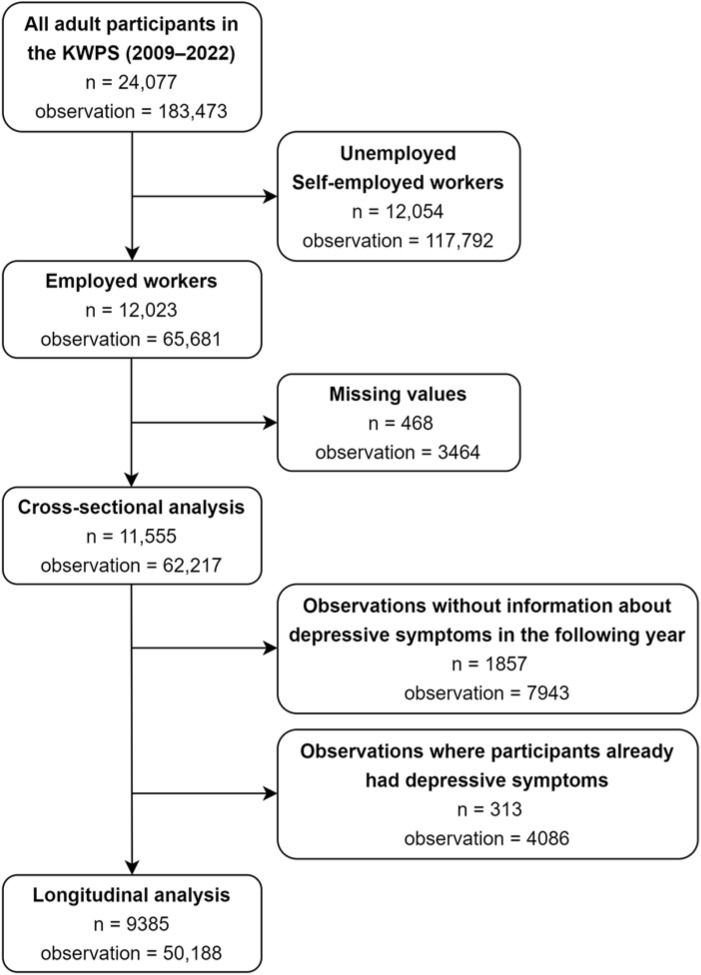
Flowchart of the selection process of study sample.

A flowchart illustrating the selection process is presented in Fig. 1. As pertinent information on PE has been collected since 2009 (Wave 4), our initial inclusion involved 24,077 adult individuals who had participated at least once from the 4th (2009) to the 17th (2022) wave of the KWPS. Subsequently, we restricted our observations to instances where each participant was a waged worker, as variables related to PE were exclusively collected for this group. After excluding observations with missing values, 11,555 individuals (representing 62,217 observations) were included in the cross-sectional analysis. For the longitudinal analysis, we first excluded observations lacking information on depressive symptoms in the subsequent year. Subsequently, we excluded observations where participants already exhibited depressive symptoms to establish a clear temporal sequence. As a result, 9385 individuals (representing 50,188 individuals) were included in the longitudinal analysis.

### Variables

#### Precarious employment

We adopted the definition of PE proposed by Kreshpaj *et al.* in their systematic review (Kreshpaj *et al.*, 2020). PE, in this context, is a multidimensional construct comprising three primary dimensions, each further delineated into multiple themes. Table 1 provides an overview of the dimensions, themes and operationalization of PE in this study.

**Table 1.** Definitions of precarious employment

Dimension	Theme	Proxy indicator (variables)	Categorization	Operationalization
**Employment insecurity**				
	Contractual temporariness	Temporary employment^a^	Permanent employment Fixed-term employment Daily employment	Duration of contract: ≥1 year Duration of contract: 1 month−1 year Duration of contract: <1 month
	Contractual underemployment	Part-time employment	a. No b. Yes	Full-time employment Part-time employment
	Contractual relation insecurity	Employment relationship	Direct employment Indirect employment Disguised employment	Direct employment Dispatch company; service company Dependent contractor
**Income inadequacy**				
	Income level	Monthly wage	a. Q4 b. Q3 c. Q2 d. Q1	Categorized into four groups according to quartile values of monthly wage for each year
**Lack of rights and protection**				
	Lack of unionization	Existence of trade union	a. Yes b. No	
	Lack of regulatory support	Coverage of social insurance^b^	a. Yes b. Borderline c. No	Coverage of all social insurances Coverage of one or two social insurances Coverage of none of the social insurances

Detailed information on the operationalization is presented in Table S1.

a This criterion corresponds to the official classification of Statistics Korea.

b National pension plan, employment insurance, industrial accidence insurance.

For the first dimension, variables such as temporary employment (permanent, fixed-term and daily employment), part-time employment (full-time and part-time employment) and employment relationship (direct, indirect and disguised employment) were selected to assess employment insecurity. The term ‘disguised employment’ refers to workers who operate within the grey area between self-employment and traditional employment. These individuals provide services for a business under a contract that differs from a typical employment contract, yet their working conditions and income are ‘dependent’ on the party with whom they have contracted. Further details on this employment type can be found at Table S1 and the report by the International Labour Organization (ILO, 2016). In the second dimension, monthly wages were categorized into four groups based on quartile values (lowest [Q1], low [Q2], high [Q3], highest [Q4]) for each year, serving as variables to gauge income inadequacy. In the third dimension, variables such as the presence of a trade union in the workplace (yes or no) and employer coverage of social insurance (yes, borderline, no) were selected to assess the absence of rights and protection in the workplace. A detailed exposition of the definition of PE is presented in Table S1.

#### Depressive symptoms

The assessment of depressive symptoms utilized the 11-item version of the Center for Epidemiological Studies-Depression scale (CES-D-11). The CES-D-11, an abbreviated form of the original 20-item CES-D, comprises questionnaires evaluating the respondents’ emotional states and vigour over the preceding week. Responses to each question were recorded as integers ranging from 0 to 3, corresponding to the following: (i) 0: rarely or none (<1 day); (ii) 1: sometimes (2–3 days); (iii) 2: often (4–5 days) and (iv) 3: most of the time (6–7 days). In this investigation, Cronbach’s alpha for the CES-D-11 ranged from 0.81 to 0.86 annually. Prior research attests to the satisfactory reliability and validity of the CES-D-11 within the general Korean population (Park *et al.*, 2022). The total score, indicative of overall depressive symptoms, spanned from 0 to 33, with a higher score signifying greater depressive symptoms. In line with established methodology from previous studies (Jeong *et al.*, 2021; Kang *et al.*, 2021; Kim *et al.*, 2023a), the total score for CES-D-11 was adjusted by multiplying it by 20 and then dividing it by 11 to align with the cut-off criteria for the 20-item version of the CES-D. Individuals with scores ≥16 were categorized as presenting depressive symptoms (Jeong *et al.*, 2021; Kang *et al.*, 2021; Kim *et al.*, 2023a).

#### Confounders

The selection of the confounders was informed by previous studies on the mental health effects of PE (Baek *et al.*, 2023; Gong and Park, 2023; Jonsson *et al.*, 2021b; Pollack *et al.*, 2022). Gender (men, women) was standardized. Age was categorized into five groups: <30, 30–39, 40–49, 50–59 and ≥60. Education level was classified as middle school or below, high school and college or above. Occupation was categorized according to the Korean Standard Classification of Occupation as blue collar, services or sales worker, or white collar. Marital status was classified as married, unmarried or other (separated, widowed, divorced). Unlike in Western countries, where the concept of de facto partnerships is socially and legally recognized, it remains uncommon in East Asian countries where traditional familial relationships are strong due to cultural influences; individuals in such partnerships were classified as unmarried. Chronic conditions were categorized as either present or absent, depending on whether the individual had been on medications for at least 6 months.

### Statistical analysis

For the descriptive analysis, we initially examined the distribution of observations during the study periods to characterize their features. Subsequently, we investigated the incidence of depressive symptoms based on study variables.

In the regression analysis, we employed generalized estimating equations (GEEs) with an exchangeable working correlation matrix to account for repeated measurements for each participant given that our primary research focus was to explore the population-averaged effects of PE on depressive symptoms. The cross-sectional association between PE elements and concurrent depressive symptoms was explored. Specifically, for each year *t*, we explored how exposure to PE elements in year *t* is associated with having depressive symptoms in year *t*. Furthermore, the longitudinal association between PE and the onset of depressive symptoms in the subsequent year was examined, assessing how exposure to PE elements in year *t* was linked to the onset of depressive symptoms in year *t* + 1. To ensure temporal sequence clarity, observations in which participants already had depressive symptoms in year *t* were excluded.

Several additional analyses were conducted. First, considering the significant impact of the COVID-19 pandemic on employment conditions and mental health in the Korean population, a sensitivity analysis excluding waves conducted from 2020 to 2022 was performed. Second, an additional analysis was conducted, treating CES-D-11 as a continuous variable, to further assess the robustness of our findings. Third, based on the operationalization of PE used in this study, we developed a scale to represent the overall level of employment precariousness for workers (Table S2). Subsequently, we investigated the relationship between the overall PE score and the onset of depressive symptoms.

Acknowledging previous evidence suggesting gender differences in the mental health effects of PE (Mendez-Rivero *et al.*, 2022; Padrosa *et al.*, 2022), analyses were conducted for the overall sample and separately for men and women. GEE models with a logit link function were employed to estimate the odds ratio (OR) and 95% confidence intervals (CIs). All statistical analyses and visualizations were performed using R software (version 4.2.3; R Foundation for Statistical Computing, Vienna, Austria). The R package ‘*geepack*’ was utilized for GEE analyses.

## Results

Table 2 displays the distribution of observations during the study period. The prevalence of temporary employment at 32.2% for fixed-term and 14.8% for daily employment. Additionally, 10.9% of participants had part-time employment, 6.3% were engaged in indirect employment and 4.4% were involved in disguised employment. About 79.0% had no trade union presence in their workplace, and 14.4% lacked social insurance coverage provided by the employer. The proportion of temporary, part-time, indirect or disguised employment was higher among women than men. Furthermore, women exhibited a tendency for lower income levels, the absence of a trade union in their workplace and a lack of coverage by social insurance.

**Table 2.** Distribution of characteristics of observations throughout the study periods

	Total	Men	Women
	*N* = 62,217	*N* = 33,176	*N* = 29,041
**Age**			
<30	9635 (15.5)	3940 (11.9)	5695 (19.6)
30−39	14,421 (23.2)	8542 (25.7)	5879 (20.2)
40−49	17,072 (27.4)	9612 (29.0)	7460 (25.7)
50−59	12,136 (19.5)	6551 (19.7)	5585 (19.2)
≥60	8953 (14.4)	4531 (13.7)	4422 (15.2)
**Education**			
Middle school or below	10,760 (17.3)	4387 (13.2)	6373 (21.9)
High school	29,973 (48.2)	17,006 (51.3)	12,967 (44.7)
College or above	21,483 (34.5)	11,783 (35.5)	9701 (33.4)
**Occupation**			
Blue collar	26,697 (42.9)	17,297 (52.1)	9401 (32.4)
Service/sales worker	11,014 (17.7)	3842 (11.6)	7172 (24.7)
White collar	24,505 (39.4)	12,037 (36.3)	12,468 (42.9)
**Marital status**			
Married	40,694 (65.4)	23,395 (70.5)	17,299 (59.6)
Unmarried	14,515 (23.3)	7474 (22.5)	7042 (24.2)
Others	7007 (11.3)	2307 (7.0)	4700 (16.2)
**Chronic condition**			
No	44,320 (71.2)	23,927 (72.1)	20,394 (70.2)
Yes	17,896 (28.8)	9249 (27.9)	8647 (29.8)
**Temporary employment**			
Permanent employment	32,985 (53.0)	20,854 (62.9)	12,131 (41.8)
Fixed-term employment	20,008 (32.2)	7786 (23.5)	12,223 (42.1)
Daily employment	9223 (14.8)	4536 (13.7)	4687 (16.1)
**Part-time employment**			
No	55,455 (89.1)	31,644 (95.4)	23,812 (82.0)
Yes	6761 (10.9)	1532 (4.6)	5229 (18.0)
**Employment relationship**			
Direct employment	55,551 (89.3)	30,017 (90.5)	25,535 (87.9)
Indirect employment	3914 (6.3)	1971 (5.9)	1943 (6.7)
Disguised employment	2751 (4.4)	1188 (3.6)	1563 (5.4)
**Monthly wage**			
Q4	15,340 (24.7)	12,701 (38.3)	2639 (9.1)
Q3	15,487 (24.9)	10,069 (30.3)	5419 (18.7)
Q2	15,669 (25.2)	5864 (17.7)	9805 (33.8)
Q1	15,720 (25.3)	4542 (13.7)	11,178 (38.5)
**Existence of trade union**			
Yes	13,072 (21.0)	8365 (25.2)	4707 (16.2)
No	49,144 (79.0)	24,811 (74.8)	24,334 (83.8)
**Coverage of social insurance**			
Yes	35,295 (56.7)	20,573 (62.0)	14,722 (50.7)
Borderline	17,935 (28.8)	10,368 (31.3)	7567 (26.1)
No	8986 (14.4)	2235 (6.7)	6752 (23.2)

Values are presented as *N* (%).

Table 3 illustrates the incidence of depressive symptoms based on study characteristics. The overall incidence of depressive symptoms was 8.3% during the study period, with a higher incidence observed among individuals engaged in temporary, part-time and indirect or disguised employment. Additionally, individuals with lower wages, without trade union in their workplace and lacking social insurance coverage displayed a higher incidence of depressive symptoms.

**Table 3.** Incidence of depressive symptoms according to study variables

	Total		Men		Women	
	Cases	Non-cases	Cases	Non-cases	Cases	Non-cases
**Age**						
<30	789 (10.6)	6687 (89.4)	347 (11.3)	2721 (88.7)	442 (10.0)	3966 (90)
30−39	837 (6.9)	11,248 (93.1)	506 (6.9)	6788 (93.1)	331 (6.9)	4460 (93.1)
40−49	978 (6.9)	13,158 (93.1)	585 (7.3)	7481 (92.7)	393 (6.5)	5677 (93.5)
50−59	792 (8.2)	8892 (91.8)	411 (7.6)	4970 (92.4)	381 (8.9)	3922 (91.1)
≥60	767 (11.3)	6040 (88.7)	333 (9.2)	3279 (90.8)	434 (13.6)	2761 (86.4)
**Education**						
Middle school or below	982 (11.7)	7442 (88.3)	358 (10.0)	3236 (90.0)	624 (12.9)	4206 (87.1)
High school	1720 (7.1)	22,562 (92.9)	957 (6.8)	13,085 (93.2)	763 (7.5)	9477 (92.5)
College or above	1461 (8.4)	16,021 (91.6)	867 (8.9)	8918 (91.1)	594 (7.7)	7103 (92.3)
**Occupation**						
Blue collar	2084 (9.7)	19,332 (90.3)	1288 (9.1)	12,872 (90.9)	796 (11.0)	6459 (89.0)
Service/sales worker	758 (8.7)	7979 (91.3)	262 (8.3)	2888 (91.7)	496 (8.9)	5091 (91.1)
White collar	1321 (6.6)	18,714 (93.4)	632 (6.3)	9478 (93.7)	689 (6.9)	9236 (93.1)
**Marital status**						
Married	2222 (6.6)	31,634 (93.4)	1279 (6.4)	18,561 (93.6)	943 (6.7)	13,073 (93.3)
Unmarried	1275 (11.4)	9923 (88.6)	696 (12.0)	5115 (88.0)	579 (10.7)	4808 (89.3)
Others	666 (13.0)	4468 (87.0)	207 (11.7)	1563 (88.3)	459 (13.6)	2905 (86.4)
**Chronic condition**						
No	2773 (7.6)	33,643 (92.4)	1541 (7.7)	18,482 (92.3)	1232 (7.5)	15,161 (92.5)
Yes	1390 (10.1)	12,382 (89.9)	641 (8.7)	6757 (91.3)	749 (11.8)	5625 (88.2)
**Temporary employment**						
Permanent	1704 (6.2)	25,673 (93.8)	1049 (6.0)	16,568 (94.0)	655 (6.7)	9105 (93.3)
Fixed-term	1471 (9.3)	14,350 (90.7)	620 (9.8)	5685 (90.2)	851 (8.9)	8665 (91.1)
Daily	988 (14.1)	6002 (85.9)	513 (14.7)	2986 (85.3)	475 (13.6)	3016 (86.4)
**Part-time employment**						
No	3600 (8.0)	41,539 (92.0)	2015 (7.7)	24,263 (92.3)	1585 (8.4)	17,276 (91.6)
Yes	563 (11.2)	4486 (88.8)	167 (14.6)	976 (85.4)	396 (10.1)	3510 (89.9)
**Employment relationship**						
Direct employment	3600 (8.0)	41,321 (92.0)	1892 (7.6)	22,984 (92.4)	1708 (8.5)	18,337 (91.5)
Indirect employment	309 (9.9)	2820 (90.1)	162 (10.0)	1452 (90.0)	147 (9.7)	1368 (90.3)
Disguised employment	254 (11.9)	1884 (88.1)	128 (13.7)	803 (86.3)	126 (10.4)	1081 (89.6)
**Monthly wage**						
Q4	714 (5.5)	12,284 (94.5)	606 (5.6)	10,253 (94.4)	108 (5.0)	2031 (95.0)
Q3	899 (7.0)	11,863 (93.0)	609 (7.3)	7791 (92.7)	290 (6.6)	4072 (93.4)
Q2	1135 (9.1)	11,345 (90.9)	491 (10.4)	4245 (89.6)	644 (8.3)	7100 (91.7)
Q1	1415 (11.8)	10,533 (88.2)	476 (13.9)	2950 (86.1)	939 (11)	7583 (89.0)
**Existence of trade union**						
Yes	650 (6.1)	10,076 (93.9)	426 (6.1)	6567 (93.9)	224 (6.0)	3509 (94.0)
No	3513 (8.9)	35,949 (91.1)	1756 (8.6)	18,672 (91.4)	1757 (9.2)	17,277 (90.8)
**Coverage of social insurance**						
Yes	2054 (7.1)	26,835 (92.9)	1177 (6.8)	16,055 (93.2)	877 (7.5)	10,780 (92.5)
Borderline	1250 (8.7)	13,080 (91.3)	751 (8.8)	7776 (91.2)	499 (8.6)	5304 (91.4)
No	859 (12.3)	6110 (87.7)	254 (15.3)	1408 (84.7)	605 (11.4)	4702 (88.6)

Values are presented as *N* (%).

Table 4 presents the cross-sectional association between temporary employment and concurrent depressive symptoms. Daily employment (OR: 1.67, 95% CI: 1.48–1.89), the lowest monthly wages (OR: 1.93, 95% CI: 1.66–2.23) and the absence of social insurance coverage (OR: 1.28, 95% CI: 1.15–1.43) were associated with an increased likelihood of having depressive symptoms in the overall sample, as well as in both men and women. Additionally, men with disguised employment were more likely to have depressive symptoms compared to those with direct employment (OR: 1.47, 95% CI: 1.18–1.84).

**Table 4.** Cross-sectional association between precarious employment and concurrent depressive symptoms

	Total	Men	Women
	OR (95% CI)	OR (95% CI)	OR (95% CI)
**Temporary employment**			
Permanent employment	1.00 (1.00−1.00)	1.00 (1.00−1.00)	1.00 (1.00−1.00)
Fixed-term employment	1.09 (0.98−1.20)	1.06 (0.90−1.25)	1.12 (0.99−1.28)
Daily employment	1.67 (1.48−1.89)	1.60 (1.32−1.94)	1.72 (1.46−2.02)
**Part-time employment**			
No	1.00 (1.00−1.00)	1.00 (1.00−1.00)	1.00 (1.00−1.00)
Yes	0.92 (0.83−1.01)	0.84 (0.68−1.03)	0.96 (0.86−1.07)
**Employment relationship**			
Direct employment	1.00 (1.00−1.00)	1.00 (1.00−1.00)	1.00 (1.00−1.00)
Indirect employment	1.00 (0.89−1.13)	1.07 (0.88−1.29)	0.96 (0.82−1.12)
Disguised employment	1.26 (1.08−1.46)	1.47 (1.18−1.84)	1.10 (0.90−1.35)
**Monthly wage**			
Q4	1.00 (1.00−1.00)	1.00 (1.00−1.00)	1.00 (1.00−1.00)
Q3	1.25 (1.10−1.43)	1.34 (1.14−1.57)	1.12 (0.89−1.43)
Q2	1.59 (1.39−1.83)	1.72 (1.43−2.09)	1.37 (1.08−1.72)
Q1	1.93 (1.66−2.23)	2.63 (2.15−3.22)	1.48 (1.16−1.89)
**Trade union**			
Yes	1.00 (1.00−1.00)	1.00 (1.00−1.00)	1.00 (1.00−1.00)
No	1.03 (0.93−1.14)	1.04 (0.89−1.21)	1.02 (0.89−1.18)
**Coverage of social insurance**			
Yes	1.00 (1.00−1.00)	1.00 (1.00−1.00)	1.00 (1.00−1.00)
Borderline	1.08 (0.99−1.18)	1.09 (0.95−1.25)	1.06 (0.94−1.20)
No	1.28 (1.15−1.43)	1.42 (1.16−1.72)	1.24 (1.08−1.41)
N of observations	62,217	33,176	29,041
N of individuals	15,555	5855	5700

OR, odds ratio; CI, confidence interval.

Models adjusted for gender, age, educational level, occupation, marital status and chronic condition.

**Figure 2.**
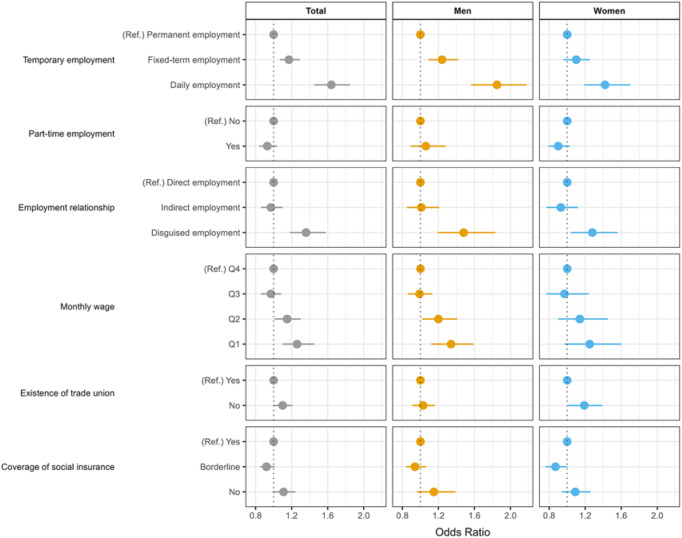
Longitudinal association between precarious employment and onset of depressive symptoms in the following year. Models adjusted for gender, age, educational level, occupation, marital status and chronic condition (OR, odds ratio; CI, confidence interval; Ref, reference).

Figure 2 shows the longitudinal association between temporary employment and the onset of depressive symptoms in the following year. In men, fixed-term (OR: 1.24, 95% CI: 1.09–1.42) or daily employment (OR: 1.84, 95% CI: 1.56–2.18), disguised employment (OR: 1.47, 95% CI: 1.19–1.83) and the lowest wages (OR: 1.34, 95% CI: 1.13–1.60) were positively associated with depressive symptom onset in the following year. In women, daily employment (OR: 1.42, 95% CI: 1.19–1.70), disguised employment (OR: 1.27, 95% CI: 1.03–1.56) and the absence of trade union (OR: 1.18, 95% CI: 1.01–1.39) were positively associated with depressive symptom onset. Detailed results of the longitudinal analyses are provided in Table S3.

Sensitivity analyses excluding waves conducted from 2020 to 2022 yielded comparable findings to the main analyses (Table S4). Additional analyses treating CES-D-11 as continuous variable produced findings that were consistent with the main analyses (Table S5). In the analysis considering overall PE level, we observed a dose–response relationship between the overall level of employment precariousness and the onset of depressive symptoms. Specifically, individuals exposed to the highest level of PE exhibited 2.3- and 1.6-fold increased odds of developing depressive symptoms in men and women, respectively (Table S6).

## Discussion

In this study, we observed that the experience of PE is associated not only with concurrent depressive symptoms but also with the onset of depressive symptoms in both male and female individuals. The overall incidence of depressive symptoms observed in this sample was comparable to that observed in the general population in Korea. Factors such as employment insecurity, insufficient income and a lack of rights and protection were identified as contributors to an increased likelihood of the onset of depressive symptoms. Consequently, proactive policy measures are warranted to improve the overall quality of employment for workers (Kang, 2023).

Our findings are consistent with prior research demonstrating the link between PE and compromised mental health among workers. As indicated in systematic reviews (Jaramillo *et al.*, 2022; Ronnblad *et al.*, 2019), previous evidence has predominantly concentrated on the type of contract. Similarly, earlier Korean studies defined PE primarily based on contract types, revealing associations between insecure employment and depressive symptoms (Han *et al.*, 2017; Jang *et al.*, 2015; Oh *et al.*, 2021). However, recent studies have emphasized that various dimensions of PE, including not only employment insecurity but also income inadequacy, as well as a lack of rights and protection, are closely linked to poor mental health (Jonsson *et al.*, 2021a, 2021b; Pollack *et al.*, 2022; Vives *et al.*, 2020). A recent cross-sectional study in Korea uncovered that workers in employment typologies with high precariousness – characterized by elevated employment insecurity, low wages with high volatility and a lack of rights and protection – were more prone to experiencing poor mental well-being (Baek *et al.*, 2023). Therefore, in alignment with this evolving perspective, our findings make a substantial contribution to the literature by demonstrating that the multidimensional aspects of PE among Korean workers are not only associated with concurrent depressive symptoms but also with their onset.

One intriguing finding in this study concerns the association between disguised employment and depressive symptoms. This employment type, alternatively termed a ‘dependent contractor’ or ‘dependent self-employed’, exists in the ambiguous realm between self-employment and waged work (Cherry and Aloisi, 2016; International Labour Office, 2017). In Korea, individuals in this employment category are required to autonomously seek and engage with clients to provide goods or services, thereby receiving income based on actual performance rather than a fixed salary. This form of employment is experiencing rapid growth, especially in conjunction with the rise of the ‘gig’ economy (Hendrickx, 2023; International Labour Office, 2017). In Korea, ongoing legal and social debates revolve around the classification of these workers as either self-employed or wage workers. Disguised employment often circumvents the application of labour laws, exposing workers to various physical and psychological workplace hazards (Kim, 2020). As observed in previous cross-sectional studies in Korea, disguised employment demonstrated a positive association with depressive and anxiety symptoms (Baek *et al.*, 2022; Won *et al.*, 2019), a correlation further affirmed in our longitudinal study.

Exposure to PE can contribute to the onset of depressive symptoms through intricate mechanisms. Initially, factors such as employment insecurity, low wages and a lack of rights and protection may heighten workers’ perceived insecurity, uncertainty and instability, consequently leading to psychological anxiety and stress (Bodin *et al.*, 2020). This observation is in line with recent studies that associate PE with elevated levels of biomarkers indicative of the stress response (Julia *et al.*, 2022; Mendez-Rivero *et al.*, 2022). Secondly, PE can subject workers to various physical, ergonomic or psychosocial workplace hazards, all of which are significant risk factors for the development of depressive symptoms (Rivero *et al.*, 2021). Thirdly, factors such as low wages and a lack of social insurances can result in material deprivation, a well-documented risk factor for the onset of depressive symptoms (Guan *et al.*, 2022; Ridley *et al.*, 2020).

In this study, the association between PE and the onset of depressive symptoms was ubiquitously observed in both men and women. However, the prevalence of PE was observed to be higher among Korean women, aligning with prior research (Baek *et al.*, 2023; Ervin *et al.*, 2023). Particularly, in East Asian countries strongly influenced by Confucian culture, a cultural perception prevails that assigns women the role of ‘caregiver’ and men the role of ‘breadwinner’. Consequently, despite possessing high educational backgrounds, women are often discouraged from pursuing professional career development, leading to their concentration in more precarious job positions (Choi, 2018). This underscores the need for the development of gender-sensitive policies aimed at improving employment quality among Korean workers.

Our findings are subject to several limitations. Firstly, the observational nature of our study prevents the establishment of a causal relationship between PE and depressive symptoms. Potential unmeasured confounders, such as prior psychiatric history, parental socio-economic position and the number of children in the household, could introduce bias into our estimations. We could not include these factors in our analyses due to a lack of information. Secondly, reliance on self-reported questionnaires for depressive symptoms introduces the possibility of measurement errors, including recall bias. While the CES-D-11 is commonly used in epidemiological studies, it was originally developed for screening purposes. Therefore, further research is warranted to confirm whether PE is associated with the onset of clinically diagnosed depressive disorders. Thirdly, limitations in the assessment of PE should be acknowledged. Specifically, in interpreting null findings between part-time employment and depressive symptoms, we were unable to distinguish between involuntary and voluntary part-time work due to lack of information. Additionally, our assessment of PE exposure did not account for other important elements, such as income volatility or vulnerability in the workplace, due to a lack of available information. Fifth, self-employed workers were excluded from our study, and detailed information on their employment conditions was not collected. Future studies should investigate employment precariousness among self-employed workers and its potential impact on their mental health in Korea (Kim *et al.*, 2023b). Sixth, information on ethnicity and migration status was not collected. South Korea is ethnically homogeneous due to its geographical isolation and unique language, with approximately 96% of the total population being native Koreans, while immigrants comprise about 3.5% of the population. However, similar to other countries, immigrants, despite constituting a relatively small proportion of the population, tend to disproportionately experience PE in Korea. Consequently, further research is necessary to investigate the current status of PE among Korean migrant workers and its mental health effects (Pollack *et al.*, 2022). Seventh, concerning the operationalization of income level, categorizing income levels based on quartile values of monthly wages may not fully reflect the ability to cover basic needs. Eighth, the potential for selection bias stemming from attrition and loss to follow-up may have impacted the estimates derived from our analysis. For instance, individuals with missing values on the onset of depressive symptoms or lacking follow-up data could exhibit high levels of employment precariousness at baseline and subsequently more prone to developing depressive symptoms (Kachi *et al.*, 2014). Consequently, such loss to follow-up could lead to an underestimation of the association between PE and the onset of depressive symptoms.

Nevertheless, the study exhibits several strengths. Firstly, a nationwide sample was employed, encompassing diverse demographics and occupations, thereby augmenting the generalizability of the findings. Secondly, prior Korean studies have focused solely on contract type or relied on cross-sectional analyses to explore the association between PE and depressive symptoms. In contrast, we contend that our investigation makes a meaningful contribution to the literature by uncovering the longitudinal association between the various facets of PE and the onset of depressive symptoms.

## Conclusion

This study elucidates the impact of various elements of PE, including employment insecurity, logarithmic wages and the lack of worker rights and protection, on the development of depressive symptoms among Korean wageworkers. The findings underscore PE as a noteworthy social determinant affecting the mental health. Consequently, proactive policy interventions are imperative to alleviate the detrimental effects of PE on mental health and to improve employment quality for Korean workers.

## Supporting information

Baek et al. supplementary material Baek et al. supplementary material

## Data Availability

The raw data are openly published at http://www.koweps.re.kr/.

## Supplementary material

The supplementary material for this article can be found at https://doi.org/10.1017/S2045796024000258.
