# Supplementary material for: Association between precarious employment and the onset of depressive symptoms in men and women: a 13-year longitudinal analysis in Korea (2009–2022)
Source: Epidemiol Psychiatr Sci. 2024 Apr 16;33:e25. doi: 10.1017/S2045796024000258 (PMC11022256; doi:10.1017/S2045796024000258)
Supplement: Baek et al. supplementary material [file S2045796024000258sup001.docx]

**Table S1.** Detailed information on the operationalization of precarious employment in this study.

| **Proxy indicator (variables)** | **Categorization (response options)** | **Detailed definition used for categorization** |
| --- | --- | --- |
| **Employment insecurity** |  |  |
| Temporary employment | a. Permanent employment b. Fixed–term employment c. Daily employment | a. Employees with a labor contract lasting one year or more, or individuals who can continue working without a specified contract duration at their discretion. b. Employees with a labor contract period lasting one month or more but less than one year. c. Employees with a work contract period of less than one month, or those who are employed on a daily basis, receiving daily wages for their work |
| Part–time employment | a. No  b. Yes | a. Full-time employment b. Working less than 36 hours per week based on the labor contract |
| Employment relationship | a. Direct employment  b. Indirect employment  c. Disguised employment | a. Employees directly hired by one employer. b. Employees for whom the contractual employer and the actual employer differ in the employment agreement. c. Employees who independently seek or engage with clients to provide goods or services and earn income (commissions, allowances, etc.) based on their work. Examples include insurance salesperson, construction machine operator, visiting teacher, golf caddie, door-to-door deliverer, quick service deliverer, loan solicitor, credit card solicitor, designated driving service worker, door-to-door salesperson, door-to-door rental equipment examiner. |
| **Income inadequacy** |  |  |
| Monthly wage | a. Q4 b. Q3  c. Q2  d. Q1 | Categorized into four groups according to quartile values of monthly wage for each year |
| **Lack of rights and protection** |  |  |
| Existence of trade union | a. Yes b. No | Presence or absence of an trade union within the workplace, regardless of union membership, |
| Coverage of social insurance^a^ | a. Yes b. Borderline c. No | a. Coverage of all social insurances^a^  b. Coverage of one or two social insurances  c. Coverage of none of the social insurances |

^a^ Coverage of national pension plan, employment insurance, industrial accidence insurance by the employer. Information regarding health insurance coverage was not collected, and given that over 99% of the Korean population is covered by health insurance (Shin *et al.*, 2022), it was not considered in the categorization of social insurance coverage.

**Table S2**. Scoring system of overall PE scale.

|  | **Score** | | | | |
| --- | --- | --- | --- | --- | --- |
| **Elements of PE** | **-2** | **-1** | **0** | **1** | **2** |
| **Employment insecurity** |  |  |  |  |  |
| Indirect employment | Indirect or disguised employment |  | Direct employment |  |  |
| Length of contract | Daily employment | Fixed-term employment | Permanent employment |  |  |
| Underemployment | Part-time employment |  | Full-time employment |  |  |
| **Income adequacy** |  |  |  |  |  |
| Monthly wage (in quartile) | Q1 (lowest) | Q2 (low) |  | Q3 (high) | Q1 (highest) |
| **Lack of rights and protection** |  |  |  |  |  |
| Existence of trade union | No |  | Yes |  |  |
| Coverage of social insurances | No | Borderline | Yes |  |  |
| **Overall score (range)** | -12 (min) – 2 (max) | | | | |
| **Categorization of PE level** | 0 ~ 2: Lowest PE group -3 ~ -1: Low PE group -6 ~ –4: High PE group -12 ~ -7: Highest PE group | | | | |

PE: precarious employment

**Table S3.** Longitudinal association between precarious employment and onset of depressive symptoms.

|  | **Total** | **Men** | **Women** |
| --- | --- | --- | --- |
|  | **OR (95% CI)** | **OR (95% CI)** | **OR (95% CI)** |
| **Temporary employment** |  |  |  |
| Permanent employment | 1.00 (1.00–1.00) | 1.00 (1.00–1.00) | 1.00 (1.00–1.00) |
| Fixed–term employment | 1.17 (1.07–1.29) | 1.24 (1.09–1.42) | 1.10 (0.96–1.25) |
| Daily employment | 1.64 (1.45–1.85) | 1.84 (1.56–2.18) | 1.42 (1.19–1.70) |
| **Part–time employment** |  |  |  |
| No | 1.00 (1.00–1.00) | 1.00 (1.00–1.00) | 1.00 (1.00–1.00) |
| Yes | 0.93 (0.84–1.04) | 1.07 (0.89–1.28) | 0.90 (0.79–1.03) |
| **Employment relationship** |  |  |  |
| Direct employment | 1.00 (1.00–1.00) | 1.00 (1.00–1.00) | 1.00 (1.00–1.00) |
| Indirect employment | 0.97 (0.86–1.10) | 1.01 (0.85–1.21) | 0.93 (0.77–1.12) |
| Disguised employment | 1.36 (1.17–1.57) | 1.47 (1.19–1.83) | 1.27 (1.03–1.56) |
| **Monthly wage** |  |  |  |
| Q4 | 1.00 (1.00–1.00) | 1.00 (1.00–1.00) | 1.00 (1.00–1.00) |
| Q3 | 0.97 (0.86–1.09) | 0.99 (0.87–1.14) | 0.98 (0.78–1.25) |
| Q2 | 1.15 (1.02–1.31) | 1.20 (1.02–1.41) | 1.16 (0.92–1.47) |
| Q1 | 1.27 (1.11–1.46) | 1.34 (1.13–1.60) | 1.27 (0.99–1.63) |
| **Trade union** |  |  |  |
| Yes | 1.00 (1.00–1.00) | 1.00 (1.00–1.00) | 1.00 (1.00–1.00) |
| No | 1.10 (1.00–1.21) | 1.03 (0.91–1.16) | 1.18 (1.01–1.39) |
| **Coverage of social insurance** |  |  |  |
| Yes | 1.00 (1.00–1.00) | 1.00 (1.00–1.00) | 1.00 (1.00–1.00) |
| Borderline | 0.92 (0.84–1.01) | 0.94 (0.84–1.06) | 0.86 (0.75–0.99) |
| No | 1.11 (1.00–1.25) | 1.16 (0.96–1.40) | 1.09 (0.94–1.26) |
| N of observations | 50,188 | 27,421 | 22,767 |
| N of individuals | 9385 | 4757 | 4628 |

OR, odds ratio; CI, confidence interval
Models adjusted for gender, age, educational level, occupation, marital status, and chronic condition.

**Table S4.** Longitudinal association between precarious employment and onset of depressive symptoms excluding survey year after 2019.

|  | **Total** | **Men** | **Women** |
| --- | --- | --- | --- |
|  | **OR (95% CI)** | **OR (95% CI)** | **OR (95% CI)** |
| **Temporary employment** |  |  |  |
| Permanent employment | Reference | Reference | Reference |
| Fixed–term employment | 1.16 (1.05–1.28) | 1.23 (1.06–1.42) | 1.10 (0.95–1.27) |
| Daily employment | 1.65 (1.45–1.88) | 1.83 (1.52–2.19) | 1.48 (1.22–1.79) |
| **Part–time employment** |  |  |  |
| No | Reference | Reference | Reference |
| Yes | 0.94 (0.83–1.05) | 1.03 (0.84–1.26) | 0.92 (0.79–1.06) |
| **Employment relationship** |  |  |  |
| Direct employment | Reference | Reference | Reference |
| Indirect employment | 0.99 (0.87–1.14) | 1.04 (0.86–1.25) | 0.96 (0.79–1.17) |
| Disguised employment | 1.46 (1.25–1.70) | 1.69 (1.35–2.11) | 1.28 (1.03–1.59) |
| **Monthly wage** |  |  |  |
| Q4 | Reference | Reference | Reference |
| Q3 | 0.94 (0.83–1.07) | 0.96 (0.83–1.11) | 1.00 (0.77–1.30) |
| Q2 | 1.16 (1.01–1.33) | 1.19 (1.00–1.42) | 1.25 (0.96–1.61) |
| Q1 | 1.29 (1.12–1.49) | 1.38 (1.15–1.67) | 1.34 (1.02–1.76) |
| **Trade union** |  |  |  |
| Yes | Reference | Reference | Reference |
| No | 1.14 (1.02–1.26) | 1.06 (0.92–1.21) | 1.24 (1.05–1.48) |
| **Coverage of social insurance** |  |  |  |
| Yes | Reference | Reference | Reference |
| Borderline | 0.92 (0.83–1.01) | 0.92 (0.81–1.05) | 0.89 (0.77–1.03) |
| No | 1.09 (0.97–1.23) | 1.14 (0.94–1.40) | 1.07 (0.91–1.25) |

OR, odds ratio; CI, confidence interval
Models adjusted for gender, age, educational level, occupation, marital status, and chronic condition.

**Table S5.** Association between precarious employment and depressive symptoms. CES-D-11 was treated as a continuous variable.

|  | **Total** | **Men** | **Women** |
| --- | --- | --- | --- |
|  | **β (95% CI)** | **β (95% CI)** | **β (95% CI)** |
| **Temporary employment** |  |  |  |
| Permanent employment | Reference | Reference | Reference |
| Fixed–term employment | -0.01 (-0.10, 0.09) | -0.06 (-0.18, 0.07) | 0.06 (-0.07, 0.20) |
| Daily employment | 0.74 (0.59, 0.89) | 0.69 (0.50, 0.89) | 0.79 (0.57, 1.01) |
| **Part–time employment** |  |  |  |
| No | Reference | Reference | Reference |
| Yes | -0.13 (-0.25, 0.00) | -0.24 (-0.48, -0.01) | -0.06 (-0.21, 0.09) |
| **Employment relationship** |  |  |  |
| Direct employment | Reference | Reference | Reference |
| Indirect employment | 0.16 (0.01, 0.30) | 0.34 (0.14, 0.54) | -0.03 (-0.24, 0.19) |
| Disguised employment | 0.29 (0.11, 0.48) | 0.61 (0.35, 0.87) | 0.03 (-0.23, 0.30) |
| **Monthly wage** |  |  |  |
| Q4 | Reference | Reference | Reference |
| Q3 | 0.18 (0.09, 0.26) | 0.22 (0.13, 0.32) | 0.18 (0.00, 0.36) |
| Q2 | 0.37 (0.26, 0.47) | 0.50 (0.36, 0.64) | 0.29 (0.10, 0.47) |
| Q1 | 0.68 (0.55, 0.81) | 1.02 (0.84, 1.20) | 0.48 (0.27, 0.68) |
| **Trade union** |  |  |  |
| Yes | Reference | Reference | Reference |
| No | 0.11 (0.04, 0.19) | 0.12 (0.03, 0.22) | 0.10 (-0.04, 0.24) |
| **Coverage of social insurance** |  |  |  |
| Yes | Reference | Reference | Reference |
| Borderline | 0.10 (0.02, 0.19) | 0.09 (-0.02, 0.20) | 0.10 (-0.04, 0.25) |
| No | 0.44 (0.31, 0.57) | 0.45 (0.24, 0.66) | 0.44 (0.27, 0.61) |

β, beta coefficient; CI, confidence interval
Models adjusted for gender, age, educational level, occupation, marital status, and chronic condition.

**Table S6.** Longitudinal association between overall precarious level and onset of depressive symptoms.

|  | **Total** | **Men** | **Women** |
| --- | --- | --- | --- |
|  | **OR (95% CI)** | **OR (95% CI)** | **OR (95% CI)** |
| **Overall PE level** |  |  |  |
| Lowest PE group | Reference | Reference | Reference |
| Low PE group | 1.13 (1.02–1.25) | 1.14 (1.01–1.30) | 1.17 (0.96–1.42) |
| High PE group | 1.39 (1.25–1.56) | 1.42 (1.23–1.65) | 1.44 (1.17–1.77) |
| Highest PE group | 1.85 (1.64–2.09) | 2.34 (1.99–2.75) | 1.65 (1.34–2.03) |

PE: precarious employment
OR, Odds Ratio; CI, confidence interval
Models adjusted for gender, age, educational level, occupation, marital status, and chronic condition.

**References**

**Shin, DW, Cho, J, Park, JH and Cho, B** (2022) National General Health Screening Program in Korea: history, current status, and future direction. *Precision and Future Medicine* **6**(1), 9-31.
